# Genomic and physiological mechanisms of high-altitude adaptation in Ethiopian highlanders: a comparative perspective

**DOI:** 10.3389/fgene.2024.1510932

**Published:** 2025-01-07

**Authors:** Wubalem Desta Seifu, Abreham Bekele-Alemu, Changqing Zeng

**Affiliations:** ^1^ Center of Cellular and Genetic Science, Henan Academy of Sciences, Zhengzhou, China; ^2^ Institute of Biotechnology, Wolkite University, Wolkite, Ethiopia; ^3^ Laboratory of Plant Molecular Biology and Biotechnology, Department of Biology, University of North Carolina Greensboro, Greensboro, NC, United States

**Keywords:** high-altitude adaptation, Ethiopian highlanders, genomics, hypoxia tolerance, physiological mechanisms, genetic diversity, comparative analysis

## Abstract

High-altitude adaptation is a remarkable example of natural selection, yet the genomic and physiological adaptation mechanisms of Ethiopian highlanders remain poorly understood compared to their Andean and Tibetan counterparts. Ethiopian populations, such as the Amhara and Oromo, exhibit unique adaptive strategies characterized by moderate hemoglobin levels and enhanced arterial oxygen saturation, indicating distinct mechanisms of coping with chronic hypoxia. This review synthesizes current genomic insights into Ethiopian high-altitude adaptation, identifying key candidate genes involved in hypoxia tolerance and examining the influence of genetic diversity and historical admixture on adaptive responses. Furthermore, the review highlights significant research gaps, particularly the underrepresentation of Ethiopian populations in global genomic studies, the lack of comprehensive genotype-phenotype analyses, and inconsistencies in research methodologies. Addressing these gaps is crucial for advancing our understanding of the genetic basis of human adaptation to extreme environments and for developing a more complete picture of human physiological resilience. This review offers a comparative perspective with Tibetan and Andean highlanders, emphasizing the need for expanding genomic representation and refining methodologies to uncover the genetic mechanisms underlying high-altitude adaptation in Ethiopian populations.

## 1 Introduction

Humans have adapted successfully to high-altitude environments around the world, enduring hypobaric hypoxia and other extreme stressors for thousands of years. High-altitude populations, including those in the Andes, Himalayas, and Ethiopian highlands, have evolved unique physiological responses to hypoxia, resulting from a reduced availability of oxygen at altitudes above 2,500 m (Fan et al., 2016; Hawks et al., 2007). In addition to hypoxia, these populations face several other challenges, such as increased exposure to solar radiation, limited food availability, low temperatures, and a unique disease ecology (interaction of diseases with ecological and environmental factors). These factors collectively influence human physiology and adaptation processes, which can vary based on demographic factors such as age, sex, and socioeconomic conditions (Beall, 2014a).

The Ethiopian Highlands, often referred to as the “Roof of Africa,” are a rugged mountainous region with elevations ranging from 2,500 to over 4,500 m above sea level. This region is home to a diverse array of populations, including the Amhara and Oromo, who have adapted to its unique ecological and environmental conditions (Asrat, 2016; Skovitina et al., 2012). The geography of the highlands is characterized by steep slopes, deep valleys, and plateaus, creating distinct microclimates and habitats. Population distribution in the highlands is influenced by these geographic features, with higher population densities found in fertile valleys and plateaus (Skovitina et al., 2012). Migration patterns have played a significant role in shaping the genetic and cultural landscape of the region, with evidence of both ancient and recent gene flow from neighboring lowland areas and beyond (López et al., 2021; Pagani et al., 2015).

The Amhara and Oromo are Ethiopia’s two largest ethnic groups, each contributing uniquely to the country’s genetic and cultural diversity. Historically, the Amhara are believed to be the descendants of early highlanders, founding the proto-Axumite and Axumite kingdoms in northern Ethiopia around the fourth century BCE (Aldenderfer, 2003). Today, a significant portion of the Amhara population resides above 2,500 m, maintaining deep-rooted ties to these high-altitude regions. Genetically, the Amhara share close affinities with the Oromo (Alkorta-Aranburu et al., 2012), yet their adaptation histories differ significantly. The Oromo, predominantly low-altitude dwellers, began migrating to high-altitude areas in southern Ethiopia approximately 500 years ago, driven largely by political factors (Quirin, 1992; Lewis, 1966). While most Oromo continue to reside in low-altitude environments, this historical migration created a unique opportunity to compare closely related ethnic groups with contrasting histories of high-altitude exposure.

Amhara’s prolonged habitation at high altitudes contrasts sharply with the Oromo’s relatively recent arrival in these environments (Aldenderfer, 2003). This divergence offers a valuable framework for examining genetic and physiological adaptations to hypoxia, as well as potential differences in adaptation mechanisms between long-term and recent highland residents. Moreover, the availability of low-altitude control populations for both ethnic groups enable more precise genomic and physiological comparisons, enhancing our understanding of high-altitude adaptation in the Ethiopian context.

**FIGURE 1:**
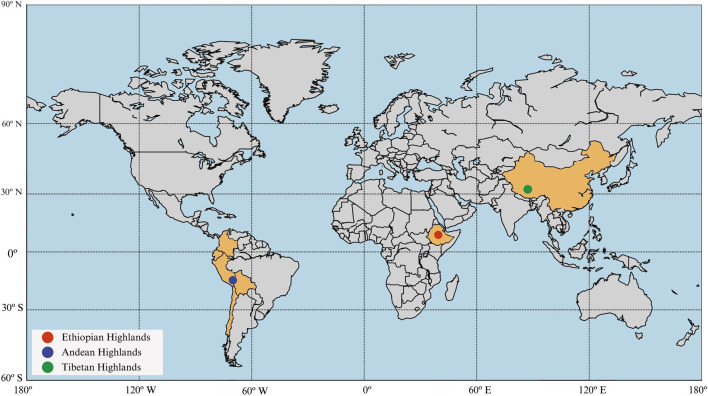
The three geographic regions of high altitude where humans have adapted. The map highlights the geographical distribution of three major high-altitude regions and their associated populations. The red dot represents the Ethiopian Highlands, the blue dot indicates the Andean Highlands, and the green dot marks the Tibetan Plateau. The map visually underscores the global diversity of high-altitude human populations and their distinct evolutionary histories.

The adaptation of high-altitude populations is not just a compelling aspect of human evolution but also provides crucial insights into our species’ resilience and biological plasticity. Recent studies indicate that the Tibetan, Andean, and Ethiopian high-altitude populations, exhibit distinct adaptive mechanisms (Figure 1). While genomic studies of Tibetan and Andean populations have shed light on their physiological and genetic responses to hypoxia, Ethiopian high-altitude adaptation remains understudied and poorly understood (Alkorta-Aranburu et al., 2012; Beall, 2007; Beall, 2000; Scheinfeldt and Tishkoff, 2010; Beall et al., 2002; Huang et al., 1992; Hoit et al., 2011; Huerta-Sánchez et al., 2013; Scheinfeldt et al., 2012; Udpa et al., 2014; Sharma et al., 2018). Given the genetic uniqueness of Ethiopian highlanders, particularly due to their history of genetic admixture (Pagani et al., 2012), understanding their adaptations is essential for a more complete picture of human high-altitude adaptation. This review aims to explore the adaptation mechanisms of Ethiopian highlanders, compare these to the adaptations seen in other high-altitude populations, and identify key research gaps that need to be addressed to further our understanding of human adaptability.

## 2 Methods

This review was conducted through a comprehensive search of relevant literature using Boolean operators (e.g., AND, OR) to combine search terms such as “high-altitude adaptation,” “Ethiopian,” “Tibetan,” “Andean,” “phenotypes,” “genotypes,” “genomics,” and “hypoxia tolerance.” The databases PubMed, Scopus, and Google Scholar were systematically reviewed to identify studies published over the past 3 decades. This time frame was chosen to capture significant advancements in genomic technologies and the resulting accumulation of high-quality data relevant to high-altitude adaptation.

The inclusion criteria for the literature included peer-reviewed original research articles, reviews, and meta-analyses that focused on the genetic and physiological mechanisms of high-altitude adaptation. Studies lacking specific relevance to Ethiopian populations or high-altitude adaptation were excluded. Extracted data were analyzed to identify key genetic markers associated with adaptation and to compare phenotypic and genomic differences across Tibetan, Andean, and Ethiopian high-altitude populations. This methodology ensured a robust and systematic approach to understanding the genetic and physiological underpinnings of human adaptation to extreme environments.

## 3 Results and comparative analysis of high-altitude adaptation mechanisms

### 3.1 Challenges of high-altitude

High-altitude environments present an array of physiological challenges that can significantly impact human health and survival. The most significant of these is hypoxia, which results from the reduced partial pressure of oxygen at higher elevations (Aldenderfer and Smith, 2014). As altitude increases, the availability of oxygen diminishes, forcing the body to undergo a range of physiological changes to maintain adequate oxygen supply to tissues. The human response to hypoxia includes increased ventilation, altered cardiovascular function, and changes in erythropoiesis, aimed at improving oxygen delivery (Beall, 2006).

Beyond hypoxia, high-altitude populations must contend with other environmental challenges, such as increased levels of solar radiation, colder temperatures, and nutritional limitations. Exposure to ultraviolet (UV) radiation is higher at greater altitudes due to reduced atmospheric filtration, which can lead to increased risks of skin damage and skin cancer (Sharma et al., 2018). Cold temperatures also pose risks such as hypothermia, particularly for populations that lack access to adequate clothing or shelter. Nutritional challenges arise from the scarcity of diverse food resources in mountainous regions, potentially leading to dietary deficiencies that impact overall health and adaptive capacity (Table 1). Disease ecology in high-altitude environments also differs significantly from lowland areas, with changes in pathogen distribution and vector dynamics that can impact immune function and disease resistance (Beall, 2014a).

**TABLE 1:** Comparison of environmental stressors across high-altitude regions.

Region	Hypoxia	UV radiation	Cold stress	Nutritional scarcity	Sources
Tibetan Plateau	Severe	High	Moderate	Moderate	Beall (2006), Jablonski (2017)
Andean Highlands	Severe	High	High	Moderate	Beall (2007), Moore et al. (1998)
Ethiopian Highlands	Moderate	High	Low	Moderate	Beall et al. (2002), Scheinfeldt et al. (2012)

These stressors collectively contribute to physiological differences between high-altitude populations and their lowland counterparts, with adaptations varying depending on a range of factors, including genetic predisposition, age, sex, and socioeconomic status. Younger individuals tend to exhibit greater plasticity and may adapt more readily to hypoxic conditions compared to older adults (Beall, 2014b; Witt and Huerta-Sánchez, 2019). Socioeconomic factors also play a critical role in determining adaptive capacity, as they influence access to resources such as nutrition and healthcare, which in turn impact physiological resilience.

A comparative analysis of the adaptation mechanisms used by Tibetan, Andean, and Ethiopian highlanders reveals both convergent and divergent evolutionary adaptations to high-altitude hypoxia (Deng et al., 2021). While all three populations have evolved mechanisms to cope with the reduced oxygen availability, their adaptive strategies differ both phenotypically and genetically. Understanding these differences is key to elucidating the processes by which human populations adapt to extreme environmental challenges.

### 3.2 The genetic background of Ethiopian populations

The genetic complexity of Ethiopian populations is shaped by their unique position at the crossroads of Africa, the Middle East, and Europe. This has resulted in a rich genetic landscape, characterized by high genetic diversity, low linkage disequilibrium, and significant non-African admixture. Genome-wide analyses reveal that this admixture, estimated to have occurred approximately 3,000 years ago, likely involved ancient migrations from the Levant and Arabia (Pagani et al., 2012; Gurdasani et al., 2015; Dobon et al., 2015).

Ethiopia also holds a central place in human evolutionary history, as evidenced by the discovery of numerous hominin fossils in the region, such as *Australopithecus anamensis*, *Australopithecus afarensis* (“Lucy”), and *Ardipithecus ramidus* (“Ardi”) (Haile-Selassie, 2001; Johanson and White, 1979; White et al., 2009). These findings emphasize the importance of Ethiopia in understanding human origins and evolutionary trajectories. Additionally, Ethiopia’s role as a migration corridor during the “Out-of-Africa” events underscores its significance in global human history. (McDougall et al., 2005; White et al., 2003).

Uniparental marker studies further illuminate the genetic history of Ethiopian populations. Mitochondrial DNA (mtDNA) analyses identify haplogroups predominantly of African origin, such as L0 and L3, alongside Eurasian lineages, including M and N. These patterns reflect both ancient African diversity and subsequent gene flow from outside the continent (Kivisild et al., 2004). Similarly, Y-chromosome analyses reveal a dual heritage, with African haplogroups like E1b1 coexisting with Eurasian haplogroups such as J1, linked to historic migrations from the Middle East (Semino et al., 2002; Passarino et al., 1998).

Ethiopian populations also exhibit genetic differences aligned with their linguistic and cultural diversity (López et al., 2021; Pagani et al., 2012). For example, Semitic-speaking Amhara and Cushitic-speaking Oromo populations show distinct genetic signatures shaped by historical migrations and localized admixture events. These variations provide a valuable framework for exploring the interplay between genetic diversity, migration, and adaptation. Moreover, the underrepresentation of Ethiopian populations in global reference genome projects highlights the need for comprehensive genomic studies to better capture the breadth of African genetic diversity (Cann et al., 2002; Genomes Project et al., 2010; The International HapMap et al., 2010; Gibbs et al., 2003; Auton et al., 2015). This has led to an incomplete picture of African genetic diversity and has hindered efforts to explore adaptive traits related to high-altitude hypoxia. Existing studies have often relied on mitochondrial DNA (mtDNA) and Y chromosome analyses, which provide only a partial view of the genetic history of the region (Gibbs et al., 2003; Auton et al., 2015). Comprehensive whole-genome studies are needed to better understand the genetic underpinnings of high-altitude adaptation among Ethiopians.

### 3.3 Adaptation mechanisms in high-altitude populations

The study of high-altitude adaptation provides a “natural laboratory” for understanding the effects of hypoxia and other stressors on human physiology (Bolton et al., 1979; Leonard and Schull Francisco Rothhammer, 1990). Advances in whole-genome sequencing have enabled researchers to identify genetic signals of adaptation and perform genotype-phenotype association studies in high-altitude populations (Tables 2, 3). By comparing the adaptation mechanisms of Tibetan, Andean, and Ethiopian high-altitude populations, we can gain insights into how different human populations have evolved unique strategies to cope with similar environmental pressures.

**TABLE 2:** Lists of high-altitude adaptations phenotypes across populations.

Phenotype	Andean	Tibetan	Ethiopian	Sources^a^
Hemoglobin	18–20 gm/dL	14–16 gm/dL	15–16 gm/dL	Alkorta-Aranburu et al. (2012), Scheinfeldt et al. (2012), Beall, (2006), Beall et al. (1998)
Oxygen saturation	92%–94%	88%–92%	92%–96%	Alkorta-Aranburu et al. (2012), Beall (2007), Scheinfeldt et al. (2012), Beall (2006)
Heart rate	70 bpm	75 bpm	70.5 bpm	Scheinfeldt et al. (2012), Beall (2006), Brutsaert et al. (1999), Brutsaert et al. (2005), Naeije (2010)
Hematocrit	54%	48%	NA	Alkorta-Aranburu et al. (2012), Beall (2007), Scheinfeldt et al. (2012), Beall (2006)
Red blood cells	5.72 × 10^6^ cells/μl	5.8 × 10^6^ cells/μl	NA	Alkorta-Aranburu et al. (2012), Scheinfeldt et al. (2012), Beall (2006), Beall et al. (1998)
Mean corpuscular volume (MCV)	90.9 fL	95 fL	NA	Beall (2007), Beall (2006), Brutsaert et al. (2019)
Lung function (volume)	87%	91%	NA	Alkorta-Aranburu et al. (2012), Brutsaert et al. (1999), Weitz et al. (2016)
Ventilation	Normal	Low	Low	Brutsaert et al. (2005), Huang et al. (1984), Zhuang et al. (1985)
Hypoxia ventilatory response (HVR)	Low	High	NA	Huang et al. (1984), Zhuang et al. (1985)
Nitric oxide (NO)	Higher than sea-level but lower than Tibetans	Elevated to increase blood flow	NA	Beall et al. (1997), Erzurum et al. (2007)

^a^ (Beall, 2006; Gilbert-Kawai et al., 2014; Garruto et al., 2003; Droma et al., 1991; Basak et al., 2021; Jansen and Basnyat, 2011; Basak et al., 2020; Bigham and Lee, 2014); NA, not available.

**TABLE 3:** Key genes implicated in high-altitude adaptation in Tibetan, Andean and Ethiopian human populations.

Population	Gene	Function/role	Sources
Tibetan	*EPAS1*	A key transcription factor involved in the hypoxia-inducible factor (HIF) pathway; regulates genes related to oxygen transport and erythropoiesis	Huerta-Sánchez et al. (2014)
	*EGLN1*	Plays a critical role in regulating the HIF pathway, which senses oxygen levels and regulates physiological responses to hypoxia	Simonson et al. (2010)
	*PRKAA2*	Protein kinase involved in cellular energy regulation under low oxygen availability, ensuring energy balance during hypoxic stress	Yi et al. (2010)
	*HMOX2*	Related to heme metabolism, it helps in reducing oxidative stress under hypoxic conditions	Simonson et al. (2012)
	*ADAM17*	Involved in the release of growth factors that help in vascular development, a critical part of adapting to high altitudes	Peng et al. (2017)
Andean	*EGLN1*	Also identified in Andeans; contributes to managing erythropoiesis, supporting adaptation via increased red blood cell counts	Bigham et al. (2010)
	*NOS2A*	Nitric oxide synthase genes are linked to increased nitric oxide production, facilitating blood flow in low-oxygen conditions	Beall (2007)
	*VEGFA*	Encodes vascular endothelial growth factor, promoting blood vessel formation to enhance oxygen delivery under hypoxic stress	Julian et al. (2009)
	*PDGFRB*	Involved in cell growth and blood vessel formation may help in vascular adaptation to hypoxic environments	Eichstaedt et al. (2015)
	*CYP17A1*	Steroidogenic enzyme; potentially linked to regulation of vascular response and blood pressure under hypoxic conditions	Bigham et al. (2009)
Ethiopian	*CBARA1*	Calcium-binding protein is involved in regulating intracellular calcium, which may help in cellular responses to hypoxia	Scheinfeldt et al. (2012)
	*THRB*	The thyroid hormone receptor that might modulate metabolic rates under hypoxia, plays a role in maintaining energy efficiency	Alkorta-Aranburu et al. (2012)
	*BHLHE41*	Basic helix-loop-helix transcription factor involved in circadian rhythm regulation, potentially aiding in sleep and energy balance at high altitude	Udpa et al. (2014)
	*ANGPTL4*	An angiopoietin-like protein that may influence vascular development and lipid metabolism, supporting adaptation in the hypoxic environment	Scheinfeldt et al. (2012)
	*EDNRA*	Encodes endothelin receptor, which is linked to vascular contraction and blood pressure regulation, crucial for hypoxia adaptation	Huerta-Sánchez et al. (2013)

#### 3.3.1 Tibetan highlanders

**FIGURE 2:**
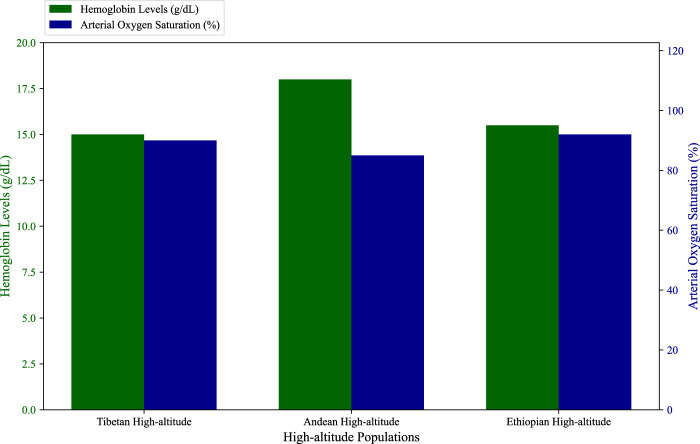
Hemoglobin concentrations and arterial oxygen saturation. The figure show comparative data of hemoglobin levels and arterial oxygen saturation across Tibetan, Andean, and Ethiopian highlanders, highlighting the distinct adaptive mechanisms employed by each group (e.g., Andean populations have higher hemoglobin levels, while Tibetan and Ethiopian populations maintain moderate levels) (Beall et al., 2002; Hoit et al., 2011; Zhuang et al., 1985; Beall and Goldstein, 1987; Beall et al., 1990; Peñaloza et al., 1963; Cheong et al., 2017).

The Tibetan Plateau, the highest permanently inhabited region in the world, is home to populations that have lived at altitudes above 4,000 m for over 30,000 years. This long history of adaptation, combined with the relative isolation of the Tibetan population, has led to the development of unique physiological and genetic adaptations to high-altitude hypoxia (Zhuang et al., 1985; Beall and Goldstein, 1987). One of the most striking features of Tibetan adaptation is the maintenance of relatively low hemoglobin levels compared to other high-altitude populations (Figure 2; Supplementary Figure S1). This is significant because excessive erythrocytosis, or an overproduction of red blood cells, can lead to cardiovascular complications, such as chronic mountain sickness (CMS), which is more common in Andean populations (Beall et al., 1990).

Instead of relying on elevated hemoglobin levels, Tibetans have adapted to hypoxia through increased nitric oxide (NO) production, which enhances vascular dilation and improves oxygen delivery throughout the body (Erzurum et al., 2007; Beall and Goldstein, 1987; Beall et al., 2012; Winslow et al., 1985). This adaptation represents a fine-tuned physiological balance between maximizing oxygen transport efficiency and minimizing the risks associated with chronic hypoxia. Tibetans also exhibit increased resting ventilation rates, which helps to improve oxygen uptake. These phenotypic traits, along with high levels of NO, suggest an adaptation strategy that minimizes physiological stress while ensuring adequate oxygen supply.

Genetically, Tibetans are distinguished by specific variants in genes involved in the hypoxia-inducible factor (HIF) pathway, including *EPAS1 (HIF-2α)* and *EGLN1 (PHD2)*. *EPAS1* has been shown to be under strong positive selection in Tibetan populations and is associated with decreased hemoglobin levels (Beall et al., 2010; Bigham et al., 2010). The unique *EPAS1* haplotype found in Tibetans is thought to have originated from introgression with Denisovan hominins, an extinct archaic human population (Xu et al., 2014). In addition to *EPAS1*, *EGLN1* variants (Table 3), such as *rs12097901* and *rs186996510*, exhibit strong positive selection in Tibetans, contributing to their ability to regulate hemoglobin levels and oxygen saturation under hypoxic conditions (Huerta-Sánchez et al., 2014). This suggests that archaic admixture may have played an important role in facilitating the adaptation of modern humans to extreme environments.

Other genes, such as *EP300* and *GCH1*, which are involved in regulating NO production and maintaining blood vessel function, have also been implicated in Tibetan high-altitude adaptation (Guo et al., 2017; Yang, 2016). These genetic adaptations collectively contribute to the unique physiological profile of Tibetan highlanders, which allows them to thrive at altitudes where oxygen availability is approximately 40% lower than at sea level.

#### 3.3.2 Andean highlanders

The Andean highlands, the second-largest high-altitude plateau in the world, are home to populations that have lived at altitudes of over 3,500 m for thousands of years. Unlike Tibetan highlanders, Andean populations have adapted to hypoxia primarily through increased hemoglobin concentration (Figure 2; Supplementary Figure S1), which enhances the oxygen-carrying capacity of the blood (Eichstaedt et al., 2014). This strategy allows Andeans to maintain adequate oxygen delivery despite the low partial pressure of oxygen at high altitudes. However, this adaptation comes with potential costs, including an increased risk of chronic mountain sickness (CMS) and pulmonary hypertension, conditions that are more prevalent among Andean highlanders compared to Tibetans (Beall, 2006; Beall et al., 1990).

The adaptive response of Andean populations is characterized by elevated levels of erythropoietin, a hormone that stimulates red blood cell production in response to low oxygen availability (Winslow et al., 1985; Julian et al., 2009). This leads to increased hemoglobin concentrations, which help compensate for the reduced oxygen levels in the environment. However, the increased red blood cell count also results in higher blood viscosity, which can contribute to complications such as CMS and pulmonary hypertension. This suggests that the Andean adaptation, while effective, may be less optimized compared to that of Tibetans, possibly due to a shorter history of exposure to high-altitude conditions.

Tibetans have *EPAS1* variants linked to lower hemoglobin levels and reduced risk of polycythemia, while Andeans have higher hemoglobin concentrations, though the selective pressure on *EPAS1* is less intense in Andeans compared to Tibetans (Beall et al., 2010; Peng et al., 2011). *EGLN1* shows strong positive selection in both populations, but the adaptive alleles are far more prevalent in Tibetans, aiding in the regulation of hemoglobin levels and oxygen saturation. These adaptive alleles are largely absent or at low frequencies in Andeans, suggesting that Andeans rely on different evolutionary mechanisms for high-altitude adaptation (Bigham et al., 2010; Eichstaedt et al., 2017).

Genomic studies have identified additional genes involved in Andean adaptation to high-altitude hypoxia, including *NOS2A, PRKAA1*, and *SENP1* (Eichstaedt et al., 2014; Zhou et al., 2013). These genes are involved in regulating nitric oxide production, energy metabolism, and erythropoiesis, respectively (Table 3). Unlike Tibetans, Andean populations do not show evidence of elevated NO production, which could otherwise help mitigate the effects of hypoxia and reduce pulmonary pressure. The distinct genetic and physiological responses of Andean highlanders highlight the diversity of human adaptation to similar environmental pressures and underscore the role of evolutionary trade-offs in shaping adaptation strategies.

#### 3.3.3 Ethiopian highlanders

Compared to Tibetan and Andean populations, Ethiopian highlanders have received less attention in the study of high-altitude adaptation. As a result, our understanding of their adaptation mechanisms is limited. Available evidence suggests that East African highlanders, particularly the Amhara and Oromo populations, do not exhibit the same increase in hemoglobin levels seen in Andean populations (Figure 2; Supplementary Figure S1). Instead, they appear to maintain hemoglobin levels like those of lowlanders, while achieving arterial oxygen saturation levels that are slightly higher than those observed in Tibetans (Beall et al., 2002; Semino et al., 2002).

Genetic studies have identified several candidate genes involved in Amhara and Oromo high-altitude adaptation. For instance, *NOS3* has been linked to increased nitric oxide production, which may help the highlanders improve oxygen delivery without increasing red blood cell counts (Pleurdeau, 2005). In addition to *NOS3*, other genes have been identified that may contribute to hypoxia adaptation in highlanders (Table 3). To illustrate, *CYP17A1*, which plays a role in steroid metabolism and may influence blood pressure regulation, has shown signals of positive selection in Amhara and Oromo populations, suggesting it could be important in maintaining cardiovascular stability under hypoxic conditions (Alkorta-Aranburu et al., 2012). *BHLHE41* (also known as *DEC2*), a gene involved in circadian rhythm and potentially influencing sleep patterns at high altitudes, has been associated with adaptation in Ethiopians, likely helping them cope with disrupted sleep patterns caused by hypoxia (Huerta-Sánchez et al., 2013). *CBARA1* (also known as *MICU1*), involved in mitochondrial calcium regulation, may play a role in enhancing cellular resilience to hypoxic stress (Udpa et al., 2014). The *VAV3* gene, which has been linked to erythropoiesis and vascular function, has also been highlighted as a potential candidate in Ethiopian adaptation due to its role in regulating the body’s response to low oxygen levels (Alkorta-Aranburu et al., 2012).

Despite these findings, the limited number of studies and inconsistencies in study design have made it difficult to draw definitive conclusions about the specific mechanisms involved. For example, some studies have used populations from the United States as low-altitude controls, rather than using genetically related East African lowlanders, which has led to conflicting results and made it challenging to compare findings across different studies (Beall et al., 2002; Hoit et al., 2011; Assefa, 2006).

### 3.4 Critical analysis of current research gaps

#### 3.4.1 Underrepresentation in genomic databases

One of the most significant challenges in studying East African high-altitude adaptation is the underrepresentation of African populations in major genomic databases, such as the 1000 Genomes Project and the HapMap Project (Semino et al., 2002; Walsh et al., 2020). Even though African populations exhibit the highest levels of genetic diversity, they are largely excluded from genomic studies that serve as the foundation for understanding human genetic variation. This underrepresentation limits our ability to identify population-specific adaptation mechanisms and to understand the full spectrum of human genetic diversity. Expanding the representation of African populations, particularly Ethiopian highlanders, in genomic studies is essential for uncovering the genetic basis of high-altitude adaptation and for providing a more complete picture of human evolutionary history.

#### 3.4.2 Inconsistent methodological approaches

Another major limitation in the study of Ethiopian high-altitude adaptation is the inconsistency in methodological approaches. Many studies have used populations from distant geographic regions as control groups, rather than using low-altitude Ethiopian populations that share a genetic background with high-altitude groups (Beall et al., 2002; Hoit et al., 2011). This inconsistency can lead to misleading results, as genetic and environmental differences between control and high-altitude populations may confound the findings. To address this issue, future studies should use genetically related low-altitude populations as controls to ensure that observed differences are due to altitude rather than other factors.

#### 3.4.3 Lack of detailed phenotype-genotype associations

Research on Tibetan and Andean populations have successfully pinpointed specific genetic loci, such as *EPAS1* and *EGLN1*, that are associated with high-altitude adaptation, enabling a deeper understanding of their physiological responses to hypoxia (Beall et al., 2010; Bigham et al., 2010). However, similar loci in the East African highlanders have yet to be definitively identified. Most current studies on Ethiopian populations lack thorough genotype-phenotype association analyses, which are critical for linking genetic variants with observed adaptive traits (Alkorta-Aranburu et al., 2012; Scheinfeldt et al., 2012). Without this data, the molecular underpinnings of adaptation to high altitude remain poorly understood. Comprehensive genomic studies that integrate physiological measurements are essential for identifying the specific loci involved in Ethiopian hypoxia adaptation and for closing the knowledge gap regarding their unique evolutionary processes.

#### 3.4.4 Insufficient physiological data collection

Current studies on Ethiopian highlanders often lack comprehensive physiological data necessary for a deeper understanding of hypoxia adaptation mechanisms. The collection of crucial physiological metrics, such as nitric oxide (NO) production, resting ventilation rates, arterial oxygen saturation, and detailed cardiovascular assessments, has been limited. These factors are essential for establishing genotype-phenotype correlations and for identifying the specific adaptive processes in Amhara and Oromo populations. Comprehensive studies examining NO production, which plays a key role in vascular dilation and oxygen delivery, have primarily focused on Tibetan populations, where elevated NO levels are noted as a major adaptive trait (Erzurum et al., 2007; Beall et al., 2010). Additionally, while arterial oxygen saturation and cardiovascular parameters have been extensively studied in Andeans and Tibetans, such measurements remain scarce in Ethiopian studies (Beall et al., 2002; Scheinfeldt et al., 2012). Future research should systematically collect these physiological data in Ethiopian highlanders to clarify their unique adaptation mechanisms and bridge the existing knowledge gap.

## 4 Future directions for Ethiopian high-altitude research

**FIGURE 3:**
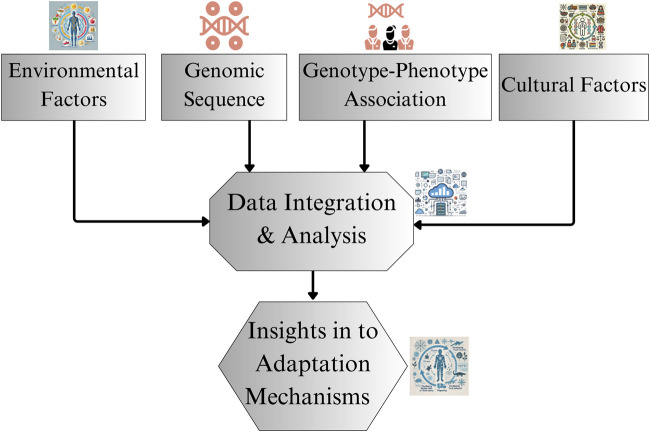
Research framework for studying Ethiopian highlander adaptations. The figure represents the proposed framework for future research, detailing components like genomic sequencing, genotype-phenotype associations, and consideration of environmental and cultural factors.

High-altitude adaptation in humans exemplifies natural selection, with populations in extreme environments evolving distinct physiological and genetic mechanisms to survive chronic hypoxia. Although research on Tibetan and Andean highlanders has provided significant insights into these mechanisms, Ethiopian highlanders present a unique case, characterized by different evolutionary pathways and physiological adaptations. To fully understand their adaptation mechanisms, a broader and more comprehensive approach is needed, one that extends beyond the oxygen transport traits typically studied in other high-altitude populations. As illustrated in Figure 3, the proposed research framework for East African highlander adaptations integrate genomic sequencing, genotype-phenotype associations, environmental factors, and cultural influences. By synthesizing this data through detailed integration and analysis, researchers can derive deeper insights into the unique adaptations of Ethiopian highlanders. Future research should focus on expanding genomic representation, conducting longitudinal and comparative studies, and broadening the scope of studies on both physiological traits and environmental influences to uncover the distinct evolutionary strategies employed by Ethiopian populations.

### 4.1 Expanding genomic representation

To address the challenges posed by the lack of genomic representation, developing a population-specific reference genome for Ethiopian populations should be a top research priority. Ethiopian populations exhibit high genetic diversity and significant admixture due to their unique history, which makes a reference genome essential for understanding not only high-altitude adaptation but also broader aspects of human evolution and disease susceptibility (Pagani et al., 2012; Gurdasani et al., 2015). The creation of a reference genome would help to correct current biases in genomic studies, which have traditionally underrepresented African populations (Author Anonymous, 2010; Need and Goldstein, 2009; Popejoy and Fullerton, 2016; Tishkoff et al., 2009). Recent research underscores the importance of including diverse population sequences in genomic databases to improve the accuracy of genetic association studies and to enhance our understanding of how different populations respond to environmental stressors (Popejoy and Fullerton, 2016). Expanding genomic data for East African populations would also contribute to global efforts aimed at capturing the full spectrum of human genetic variation (Pagani et al., 2015).

### 4.2 Longitudinal and comparative studies

Longitudinal studies tracking Ethiopian highlanders and lowlanders over time are crucial for understanding the physiological changes associated with prolonged high-altitude exposure. Such studies can elucidate whether certain adaptive traits, like hemoglobin concentration and oxygen saturation levels, remain stable or change over time in response to continuous hypoxia. Research on Andean and Tibetan populations have demonstrated the importance of longitudinal designs in assessing adaptive traits and their maintenance over generations (Beall, 2007; Moore et al., 2001). Comparative studies between the Amhara and Oromo populations could reveal differences in adaptive strategies due to factors like the length of high-altitude habitation or genetic admixture with non-African populations, which could influence traits such as nitric oxide production and oxygen transport efficiency (Alkorta-Aranburu et al., 2012; Scheinfeldt et al., 2012). Standardizing the physiological metrics used, such as hemoglobin levels and NO production, is key for drawing meaningful comparisons across studies, a challenge noted in cross-population analyses of high-altitude adaptation (Beall, 2014b).

### 4.3 Detailed genotype-phenotype association studies

Detailed genotype-phenotype association studies are essential to pinpoint genetic variants linked to adaptive traits in East African highlanders. Whole-genome sequencing (WGS) of both highland and lowland East African populations, combined with physiological assessments, provides a robust method for identifying loci associated with hypoxia tolerance. WGS has been successfully used in other high-altitude populations to detect dense genetic variations selected for in hypoxic environments (Bigham et al., 2010; Yi et al., 2010). However, one challenge remains in associating these genomic variants with specific adaptive phenotypes, particularly when many of the adaptive changes occur in non-coding regions of the genome (Huerta-Sánchez et al., 2013; Huerta-Sánchez et al., 2014).

Additionally, exploring how genetic variants interact with environmental factors, such as dietary habits and altitude-related stressors, could clarify how admixture and local environmental conditions influence adaptive traits (Scheinfeldt et al., 2012). Integrating omics technologies, such as transcriptomics and metabolomics, into genotype-phenotype studies would further illuminate the molecular mechanisms behind observed adaptations (Xu et al., 2011). Consolidating genotype-phenotype interactions in future studies will help bridge the gap between genomic data and their corresponding physiological effects, enabling a more comprehensive understanding of high-altitude adaptation.

### 4.4 Integrating environmental and cultural influences

High-altitude adaptation does not occur in isolation—environmental and cultural factors play a central role in shaping physiological responses. Socioeconomic status, diet, lifestyle, and healthcare access are all factors that can affect hypoxia tolerance. For example, dietary diversity and nutritional status may affect an individual’s ability to produce red blood cells or maintain optimal nitric oxide levels. Future research should adopt a multidisciplinary approach that integrates genetic, environmental, and cultural perspectives to provide a more holistic understanding of Ethiopian high-altitude adaptation.

High-altitude adaptation does not occur in isolation; environmental and cultural factors significantly influence physiological responses to hypoxia. Elements such as socioeconomic status, diet, lifestyle, and access to healthcare have been shown to affect an individual’s capacity to tolerate low oxygen environments. For instance, dietary diversity and adequate nutrition can influence the body’s ability to produce red blood cells and regulate nitric oxide levels, which are crucial for oxygen transport and vasodilation in hypoxic conditions (Beall, 2007). Studies have demonstrated that cultural practices, such as traditional dietary habits or high-altitude herding lifestyles, impact on how populations adapt physiologically (Julian et al., 2009). Future research should adopt a multidisciplinary approach that integrates genetic data with environmental and cultural perspectives to provide a more holistic understanding of high-altitude adaptation in Ethiopian populations. This comprehensive view will be crucial for understanding the full range of factors contributing to their resilience at high altitudes.

### 4.5 Expanding the scope of phenotypic studies

One of the key limitations in current high-altitude research is the narrow focus on phenotypes related to oxygen transport, such as hemoglobin levels, arterial oxygen saturation, and blood flow. While these are crucial aspects of adaptation, the full range of adaptive phenotypes at chronic hypoxia has not been systematically explored. Techniques and sampling conditions make it challenging to accurately measure diverse physiological traits that may collectively affect reproductive fitness. For instance, little is known about the metabolic, cardiovascular, and reproductive systems’ responses under hypoxic conditions, and their roles in adaptation remain underexplored.

Future studies should broaden their focus to include metabolic responses, immune function, and other physiological mechanisms that may maximize oxygen utility and overall fitness. Moreover, most phenotypic data have been derived from Tibetan and Andean populations and applying these results to Ethiopian highlanders may be inappropriate. It is essential to develop standardized methodologies for capturing a wider array of phenotypic traits in underrepresented populations like Ethiopians to ensure a more comprehensive understanding of adaptation mechanisms.

One of the main limitations in current high-altitude research is the narrow focus on oxygen transport-related phenotypes, such as hemoglobin levels, arterial oxygen saturation, and blood flow. While these traits are fundamental to high-altitude adaptation, other adaptive phenotypes, particularly those related to metabolism, cardiovascular health, and reproductive systems, have not been thoroughly explored (Beall, 2007; Moore, 1985). The physiological mechanisms that support overall fitness and reproductive success under chronic hypoxia remain understudied due to challenges in sampling techniques and environmental conditions (Storz et al., 2010). For instance, research has only recently begun to explore metabolic and immune system adaptations in high-altitude populations (Levett et al., 2011).

Future studies must broaden their focus to include diverse physiological responses, such as metabolic efficiency and immune function, which may play equally crucial roles in maximizing oxygen utility and overall survival at high altitudes (Julian et al., 2009). Given that most phenotypic data have been derived from Tibetan and Andean populations, applying these findings to Ethiopian highlanders without appropriate studies may lead to inaccurate conclusions (Scheinfeldt et al., 2012). Developing standardized methodologies to capture a broader range of phenotypic traits in underrepresented populations like Ethiopian highlanders is critical to achieving a more holistic understanding of adaptation mechanisms.

### 4.6 Standardization of methodologies

Standardizing methodologies for the collection and analysis of physiological and genetic data is crucial for meaningful comparisons between studies on high-altitude adaptation. Inconsistencies in measuring key physiological metrics, such as hemoglobin concentration, arterial oxygen saturation, and nitric oxide (NO) production, have hindered the comparability of findings across different studies (Beall, 2007; Moore, 1985). Ensuring that common metrics are measured using consistent and validated protocols will enable researchers to better understand the unique adaptation mechanisms among Ethiopian highlanders compared to other high-altitude populations (Scheinfeldt et al., 2012; Simonson et al., 2010). For example, standardized procedures in assessing oxygen transport and blood flow parameters have improved comparative studies of Tibetan and Andean populations and applying similar approaches to Ethiopian populations would yield clearer insights into their distinct physiological responses (Erzurum et al., 2007; Julian et al., 2009).

## 5 Limitations

While this review highlights the unique genetic adaptations of Ethiopian populations, it is important to note several limitations in the current body of research. One significant limitation is the underrepresentation of Ethiopian populations in global genomic databases, which results in an incomplete understanding of African genetic diversity. Most studies have focused on a limited number of Ethiopian populations, which may not fully capture the genetic variation present across the country.

Additionally, inconsistencies in methodologies between studies, such as differences in sample sizes, genotyping platforms, and data analysis approaches, may lead to challenges in comparing results across studies. These methodological variations can introduce bias or limit the reproducibility of findings.

Another limitation is the reliance on uniparental markers (e.g., mtDNA and Y-chromosome) in many genetic studies of Ethiopian populations. While these markers provide valuable insights into maternal and paternal lineages, they only offer a partial view of the genetic history of the region. Comprehensive whole-genome studies are needed to better understand the genetic underpinnings of adaptation in Ethiopian populations.

Finally, while this review aims to provide a broad overview of genetic and physiological adaptations, the focus on high-altitude populations has limited the exploration of other environmental pressures that may shape genetic diversity in Ethiopia. Future studies should consider the role of other ecological factors, such as disease exposure, diet, and climate variability, in shaping the genetic landscape of Ethiopian populations.

## 6 Conclusion

Ethiopian high-altitude populations provide an important yet underexplored perspective on human adaptation to extreme environments. Unlike Tibetan and Andean populations, Ethiopian highlanders exhibit unique adaptive strategies that are influenced by their complex genetic admixture and long history of exposure to diverse altitudes. Despite their importance, the specific mechanisms underlying Ethiopian adaptation remain poorly understood due to inconsistent methodologies, lack of comprehensive data, and underrepresentation in genomic databases.

Future research should focus on expanding genomic representation, using consistent study designs, conducting longitudinal analyses, and performing detailed genotype-phenotype association studies. By incorporating environmental and cultural contexts, we can not only enhance our understanding of Amhara and Oromo high-altitude adaptations but also gain broader insights into human resilience, the interplay between genetic predisposition and environmental factors, and evolutionary biology. Addressing the current research gaps will ultimately help provide a more complete picture of how diverse human populations have adapted to the challenges presented by high-altitude environments.
