# Supplementary material for: Genomic and physiological mechanisms of high-altitude adaptation in Ethiopian highlanders: a comparative perspective
Source: Front Genet. 2025 Jan 7;15:1510932. doi: 10.3389/fgene.2024.1510932 (PMC11747213; doi:10.3389/fgene.2024.1510932)
Supplement: Supplementary file 1 [file Table1.docx]

**Figure S1: Comparative Hemoglobin Levels Across Highland Populations** - This bar chart compares the hemoglobin concentrations in Tibetan, Andean, and Ethiopian high-altitude populations. Andean populations show significantly higher levels, reflecting their adaptation strategy to hypoxia through increased red blood cell count, unlike Tibetans and Ethiopians
