# Supplementary figures and images for: Genomic and physiological mechanisms of high-altitude adaptation in Ethiopian highlanders: a comparative perspective
Source: Front Genet. 2025 Jan 7;15:1510932. doi: 10.3389/fgene.2024.1510932 (PMC11747213; doi:10.3389/fgene.2024.1510932)

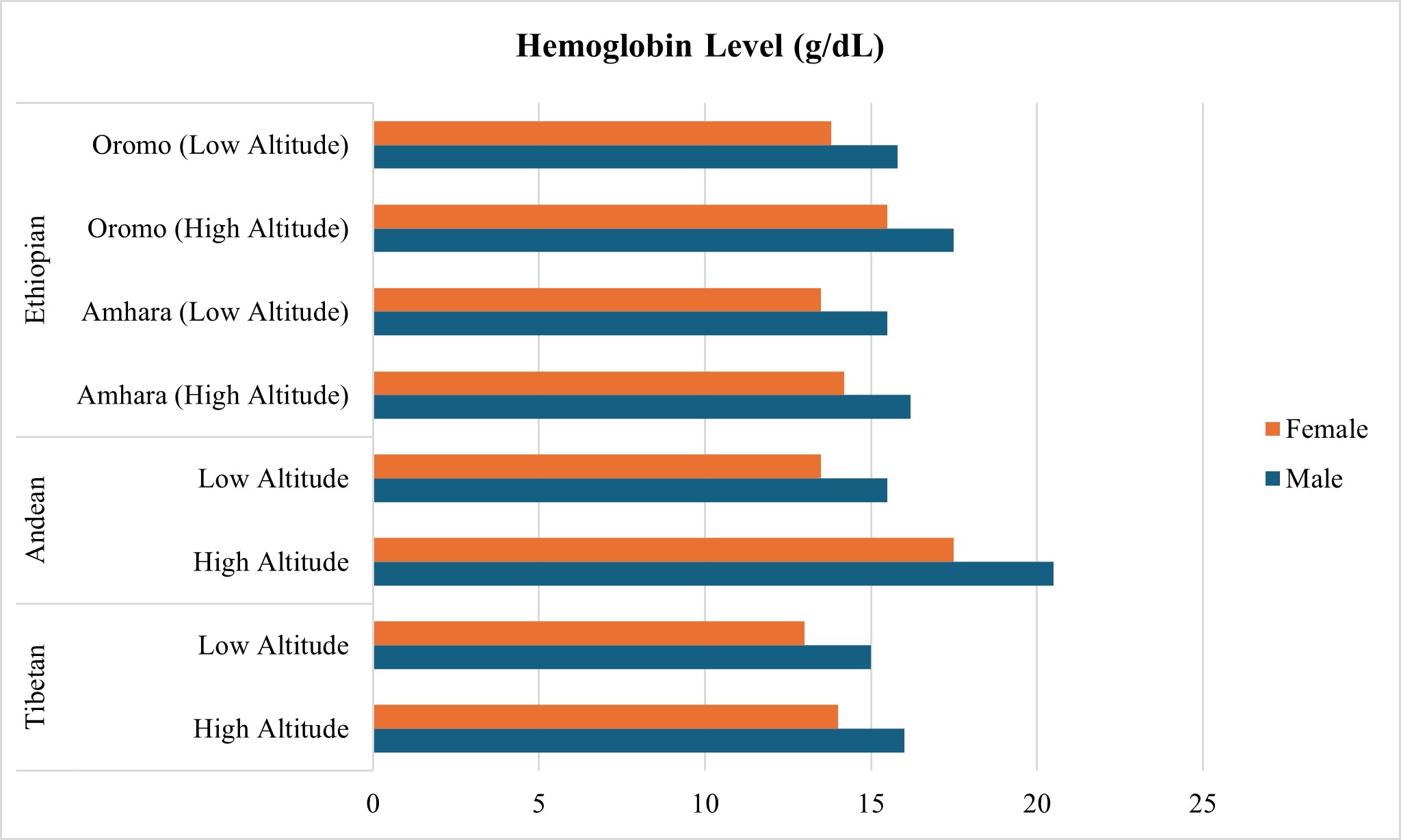

Supplement: Supplementary file 2 [file Image1.jpg]
